# Molecular Mechanisms behind Obesity and Their Potential Exploitation in Current and Future Therapy

**DOI:** 10.3390/ijms25158202

**Published:** 2024-07-27

**Authors:** Michał Nicze, Adrianna Dec, Maciej Borówka, Damian Krzyżak, Aleksandra Bołdys, Łukasz Bułdak, Bogusław Okopień

**Affiliations:** Department of Internal Medicine and Clinical Pharmacology, Faculty of Medical Sciences, Medical University of Silesia in Katowice, Medyków 18, 40-752 Katowice, Polandaboldys@sum.edu.pl (A.B.); bokopien@sum.edu.pl (B.O.)

**Keywords:** appetite, energy expenditure, gastrointestinal tract hormones, gut–brain axis, incretins, microbiome, obesity, retatrutide, semaglutide, tirzepatide

## Abstract

Obesity is a chronic disease caused primarily by the imbalance between the amount of calories supplied to the body and energy expenditure. Not only does it deteriorate the quality of life, but most importantly it increases the risk of cardiovascular diseases and the development of type 2 diabetes mellitus, leading to reduced life expectancy. In this review, we would like to present the molecular pathomechanisms underlying obesity, which constitute the target points for the action of anti-obesity medications. These include the central nervous system, brain–gut–microbiome axis, gastrointestinal motility, and energy expenditure. A significant part of this article is dedicated to incretin-based drugs such as GLP-1 receptor agonists (e.g., liraglutide and semaglutide), as well as the brand new dual GLP-1 and GIP receptor agonist tirzepatide, all of which have become “block-buster” drugs due to their effectiveness in reducing body weight and beneficial effects on the patient’s metabolic profile. Finally, this review article highlights newly designed molecules with the potential for future obesity management that are the subject of ongoing clinical trials.

## 1. Introduction

The rates of obesity and overweight have increased globally over the past half-century and continue to grow, posing a worldwide challenge to public healthcare systems [1]. It is estimated that over 60% of adults in some of the top developed countries, such as the United States, Canada, or the United Kingdom, are at least overweight [2]. To make matters worse, the human coronavirus pandemic (COVID-19) has accelerated these rising trends lately [3], especially in the pediatric population [4].

According to the World Health Organization (WHO), the diagnosis of obesity and overweight in adults is based on the body mass index (BMI), which is calculated by dividing weight in kilograms by height in meters squared. The threshold for obesity is set at BMI ≥ 30.0 kg/m^2^, and that for overweight at BMI ≥ 25.0 kg/m^2^ [5]. In children up to 5 years of age, the diagnosis is based on the WHO Child Growth Standards—obesity is weight-for-height > 3 standard deviations (SD) above the median, and overweight is weight-for-height > 2 SD above median. However, in 5-year-old or older children, obesity and overweight are defined as BMI-for-age > 2 SD and > 1 SD above the WHO Growth Reference median, respectively [6].

There are many different factors contributing to obesity’s development, but a long-lasting disruption in energy balance with caloric intake exceeding expenditure is considered to be a major cause. The foregoing may be influenced by lifestyle (e.g., preferred dietary habits, diet quality, or physical inactivity), which is determined by various social, psychological, behavioral, economic, or environmental factors [7]. Other plausible mechanisms affecting one’s propensity for obesity include family genetics and epigenetic modifications, as well as interactions between the central nervous system (CNS) and gastrointestinal (GI) tract, together with gut microbiome alterations, modulating appetite control, which have been widely discussed in the recent literature [8,9,10,11].

Genetic susceptibility to obesity concerns only a fraction of the population and is the resultant of rare mutations in single genes (e.g., *LEPR*, *PCSK1*) or chromosomal abnormalities (e.g., Prader–Willi syndrome). These rare, high-risk genetic variations are linked to obesity, which typically has an early onset and proceeds with extreme weight gain. On the other hand, for the majority of obese patients, the background is multifactorial and polygenic, and that is why traditional predictors, such as parental history of obesity and childhood obesity, outperform genetic predictors in this group [12]. Although there is an increasing interest in using genetic testing in the general population in order to identify individuals at risk of developing obesity, for most of the identified loci scientists do not yet know their exact role and do not fully understand the underlying mechanisms. Despite the difficulties in validating causative mutations and variants, gene discovery studies in the field of both monogenic and polygenic obesity have revealed shared genetic and biological underpinnings, pointing to a key role of the brain in the control of body weight [13]. Such knowledge has led to the first attempts at obesity treatment tailored to patient genotype, which is mentioned further in this article. Moreover, even though obesity coincides with type 2 diabetes mellitus (T2DM), and over 80% of patients with T2DM also have obesity, there are still some lean, but not healthy, individuals with the “metabolically obese, normal-weight” phenotype [14]. Interestingly, when T2DM occurs in patients with normal weight, mortality rates are higher than in overweight or obese people. As it turns out, such divergent features, also called “diabesity discordance”, may be dependent on genes that are potentially involved in the molecular mechanisms leading to protection against T2DM in obesity, which may represent potential targets for precise therapeutic approaches [15].

As far as epigenetic mechanisms are concerned, DNA methylation, histone modifications, and RNA-mediated processes may be associated with obesity or related phenotypes in human tissues that are relevant to metabolism, such as adipose tissue, skeletal muscles, and the liver. Aging, as well as some environmental factors, including exercise and diet, contributes to the epigenetic variability seen in patients suffering from obesity [16]. Surprisingly, histone deacetylase-responsive gene expression has a direct impact on pancreatic B-cell proliferation and, along with reduced expression of an imprinted gene network including neuronatin, may be connected with overgrowth polyphenism and cause an obese “on” state, which has been tested in isogenic mice models [17].

There are several novel proteins (e.g., soluble urokinase plasminogen activator receptor, fibroblast growth factor 23, thrombospondin-2, proteinase-3, or interleukin-18) and metabolites (e.g., carnitines, indole 3-propionate, cinnamoylglycine, threonine, or eicosapentaenoic acid) with emerging functions in obesity mechanisms, especially involved in those connected with satiety and appetite, energy balance, adipose tissue metabolism, and inflammation, which were identified as possible molecular fingerprints of susceptibility to BMI gain in young adults with over two decades of follow-up into mid-life [18]. Metabolomic signatures, meanwhile, allow us to identify the healthy obese and lean individuals with abnormal metabolomes and, thus, differentiate health outcomes in these groups [19]. Similarly, lean individuals with an obesity-related metabolome have an increased risk of obesity-related diseases, such as T2DM, in comparison to lean individuals with a healthy metabolome [20].

Accumulation of excess body fat, especially in the visceral adipose tissue, may induce a vast number of comorbidities. Some of them, such as the already-mentioned T2DM, along with hyperlipidemia or hypertension, comprise metabolic syndrome [21], eventually increasing cardiovascular risk and, hence, potentially resulting in ischemic heart disease and ischemic or hemorrhagic stroke [22]. Of no less significance are other consequences of obesity—chronic kidney disease [23], metabolic dysfunction-associated steatotic liver disease (MASLD) [24], sleep-related breathing disorders with obstructive sleep apnea and obesity hypoventilation syndrome [25], obesity-related cancers (e.g., esophageal or colorectal adenocarcinomas) [26], osteoarthrosis [27] and, last but not least, mental health disorders such as depression or anxiety [28].

**Figure 1:**
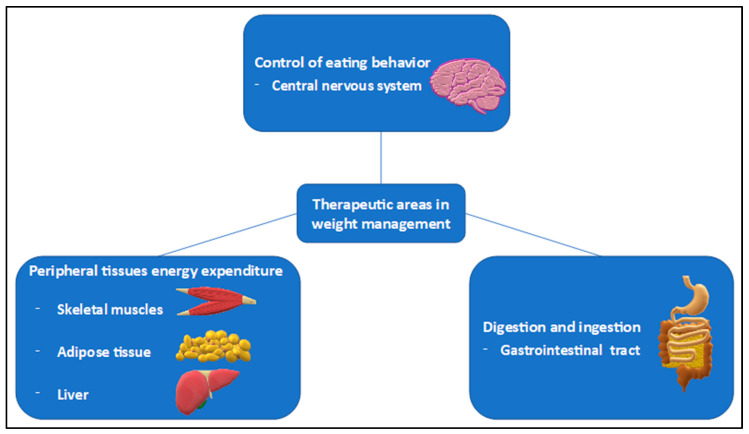
The pillars of weight management.

All of the abovementioned conditions in obese patients with cardiovascular–kidney–metabolic burden [29] lead to reduced life expectancy [30] and deterioration in quality of life [31]. It is therefore essential to focus on obesity prevention from the youngest age and provide appropriate treatment in cases of diagnosed obesity. Currently, we are gaining access to new therapies approved in weight management. Unfortunately, despite their efficacy, we still observe that a few patients fail to respond to typical treatment or show adverse effects that render the use of incretin mimetics impossible (e.g., acute pancreatitis). Therefore, in this paper, we highlight the molecular mechanisms behind the development of obesity, along with their possible utilization as the targets in the current and potential future pharmacotherapy in adults (Figure 1).

## 2. Central Nervous System (CNS)

### 2.1. Satiation, Satiety, and Hunger

Appetite regulation takes place at the level of both central and peripheral mechanisms, which should be responsible for the body’s appropriate response to the provision of food. In order to better understand the mechanisms of obesity’s development, it is essential to start with explaining concepts such as satiation, satiety, and hunger, which drive the cycle of eating and fasting. Satiation is a factor that determines the size of a meal and the moment of its completion. Its presence may be associated with the appearance of sensations such as fullness, or even nausea [10]. Once satiation is achieved, satiety begins, and during this period the feeling of hunger is inhibited [32]. In turn, as satiety disappears, hunger gradually returns into play and acts as a motivating factor to obtain food. The accompanying physical sensations include dull abdominal pain, stomach growling, and weakness [10].

### 2.2. Central Regulation of Food Intake

At the central level, the hypothalamus and the brain stem play a primary role in the mechanism of appetite control. In response to peripheral stimulation of mechano- and chemoreceptors via afferent conduction, signals are transmitted through the vagus nerve to the brain stem. They are then sent to the structures of the hypothalamus (dorsomedial, paraventricular, and arcuate nuclei), amygdala, and stria terminalis [33]. Within the arcuate nucleus itself, there can be distinguished two types of neurons acting in opposition to each other. The first ones, with an appetite-stimulating (orexigenic) effect, express neuropeptide Y (NPY) and agouti-related peptide (AgRP). The other group causes appetite suppression (anorexigenic effect) by expressing proopiomelanocortin (POMC) and cocaine- and amphetamine-regulated transcript (CART) peptide [33,34].

The action of both neuronal groups may be mutually blocked—AgRP/NPY by β-endorphin, and POMC/CART by gamma-aminobutyric acid (GABA). Additionally, at the level of the paraventricular nucleus, both types of signals from the hypothalamus are initiated by connection with melanocortin-3 and -4 receptors (MC3R and MC4R, respectively), as well as by changes in leptin levels. As a result of this, the following anorexigenic hormones are produced: corticotropin-releasing hormone (CRH), thyrotropin-releasing hormone (TRH), oxytocin, and brain-derived neurotrophic factor (BDNF) [35]. At this point, it is worth mentioning the hormones produced in the intestines, which influence multiple regions of the brain in addition to their peripheral effects—namely, ghrelin (gastric hormone stimulating hunger by AgRP/NPY neuron activation) and some anorexigenic enterohormones such as peptide YY (PYY), cholecystokinin (CCK), or glucagon-like peptide 1 (GLP-1) [36]. The latter also functions centrally as a neurotransmitter by influencing body weight, glucose, and energy metabolism, but also diminishing behaviors related to the food reward system and selectively promoting a reduction in high-fat diet consumption [34,37]. Moreover, not only the receptors but also the intestinal peptides themselves are expressed in the brain; CCK and GLP-1, for instance, are synthesized in discrete cell groups of the brain stem [38]. The detailed description of the peripheral effects of enterohormones is more widely discussed in the later section dedicated to the GI tract.

As for leptin, it is produced in white adipose tissue, acts at the level of the arcuate nucleus, and causes the activation of neurons that express POMC/CART by inhibiting the formation of AgRP/NPY. Additionally, it also has an inhibitory effect on the lateral hypothalamus and mesolimbic dopaminergic neurons, and it activates the BDNF cascade, thus suppressing appetite and increasing energy expenditure. Similar to leptin, insulin causes a decrease in appetite as well, which is associated with a reduction in NPY expression and an increase in POMC within the arcuate nucleus [33].

Regulation at the brain level also takes place through response to signals related to the deficiency/excess of specific nutrients. Intermediate products of intracellular fatty acid metabolism, like long-chain fatty acyl-CoA (LCFA-CoA) molecules, constitute a signal of satiety occurring when free fatty acids are in abundance. An increased amount of LCFA-CoA in the hypothalamus has anorexigenic effect and leads to weight loss and inhibition of NPY gene expression [39].

Moreover, nutrients may indirectly regulate the activity of the vagus nerve by influencing the secretion of gut peptides and neurotransmitters from enteroendocrine cells, like glucose stimulating the release of serotonin, increasing the exposure of receptors to it and thereby activating satiety signaling [40,41].

Compounds that may influence the inhibition of food intake also include endogenous mediators such as cytokines, e.g., interleukin-6 (IL-6) or tumor necrosis factor-α (TNF-α) [42].

**Figure 2:**
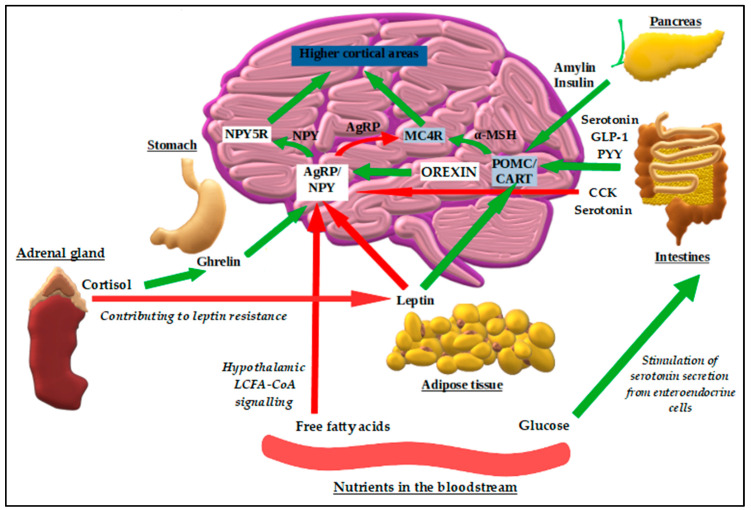
The regulation of food intake at the level of the CNS. Green arrows mean “stimulation”, whereas the red ones stand for “inhibition”. The abbreviations are defined as follows: α-MSH—α-melanocyte-stimulating hormone; AgRP—agouti-related peptide; CART—cocaine- and amphetamine-regulated transcript; CCK—cholecystokinin, GLP-1—glucagon-like peptide 1; LCFA-CoA—long-chain fatty acyl-CoA; MC4R—melanocortin-4 receptor; NPY—neuropeptide Y; NPY5R—NPY receptor type 5; POMC—proopiomelanocortin; PYY—peptide YY.

The central regulation of food intake is illustrated in Figure 2.

### 2.3. Non-Homeostatic Appetite Control

The term “hedonistic eating” refers to the consumption of food that is not related to the body’s energy needs, but rather the reward being associated with the intake of meals that are usually high in calorie content and have improved palatability [43]. This form of nutritional control is also called signal-reactive. The mesolimbic system is responsible for this mechanism. During fasting accompanied by low glucose concentrations, glutamate and orexin neurons are activated in the lateral hypothalamus area, which, in turn, stimulates dopaminergic neurons within the ventral tegmental area and increases food consumption in a hedonistic mechanism [44,45]. Furthermore, hypothalamic neurons producing orexin (hypocretin) are involved in some health-related behaviors, like narcolepsy type 1, wakefulness, drug seeking, arousal, or foraging behavior. The reactivity of hedonistic eating mechanisms is also enhanced by cortisol produced by the adrenal glands [41].

### 2.4. Disorders Underlying Obesity

In obese patients, data show the formation of low-grade inflammation within the CNS, as well as increased resistance to leptin [46]. In rodent studies, inflammation was observed in areas of the hypothalamus involved in the energy balance within just a few days of a high-fat diet. This was related to increased levels of fatty acids and their penetration through the blood–brain barrier, as well as induction of the inflammatory reaction of microglia, macrophages and, thus, local inflammation in the arcuate nucleus, paraventricular nucleus, and median eminence [47]. Additionally, studies on mice have shown that the activation of c-Jun N-terminal kinase 1 (JNK1) and inhibitor of nuclear factor kappa-B kinase 2 (IKK2) plays a key role in the development of insulin and leptin resistance in AgRP neurons and, therefore, increased appetite and obesity development [48].

Alternatively, excessive activation of the STAT3/SOCS3 pathway may be responsible for the development of the abovementioned insulin and leptin resistance [48]. At the same time, when POMC and AgRP neurons become resistant to insulin as a consequence of a high-fat diet, neurons expressing SF-1 within the ventromedial nucleus show increased sensitivity, which, in turn, translates into impaired glutamatergic innervation of POMC [49]. The phenomenon of differential hormone sensitivity is called selective hormone resistance [48]. Returning to rodent studies, it has been observed that, in individuals fed a high-fat diet, the ability of ghrelin to stimulate food intake by influencing AgRP/NPY in the arcuate nucleus is impaired. This phenomenon is not observed in the paraventricular nucleus, where it still exerts an orexigenic effect. Finally, it is worth mentioning another adaptive mechanism that develops in obesity, namely, the reduced ghrelin concentration in this group [33,50].

### 2.5. Dysregulation of the Reward System

In times of the ever-growing problem of obesity, the mechanism of hedonistic eating has become the subject of many studies. Several theories support its important role in changes in the development of obesity. The first one assumes an increased reward response in obese patients to high-calorie, palatable products, which stimulates this group to excessive consumption in order to experience pleasure [33,51]. Over time, as a result of chronic exposure to a stimulus in the form of high-calorie food, the reward system shows reduced sensitivity (downregulation). Then, in order to achieve the same effect as before, obese people must consume larger amounts of specific foods, which further fuels the cycle of energy oversupply [43]. Other causes of increased weight are linked to cravings for specific groups of products, predominantly containing large amounts of sugar, salt, and fats, resembling to some extent a form of addiction [33,52]. Dysregulation of the reward system is also triggered by insulin and leptin resistance observed in obese individuals.

Additionally, due to the connection with the areas of the brain responsible for supervision and decision-making, obese patients have an impaired ability to control impulses, which makes them unable to resist eating unhealthy food despite being aware of its harmful impact [53].

Binge-eating disorder (BED) is a mental disorder characterized by episodes in which large amounts of food are consumed uncontrollably in a short period of time, and it often co-occurs with obesity. Aberrant reward processing connected with mesocorticolimbic pathways, i.e., hypersensitivity to food rewards and hyposensitivity to general ones, contributes to both of these disorders [54]. Moreover, abnormalities occurring in BED can be compared to those observed in people addicted to psychoactive substances—they involve changes in the prefrontal and orbitofrontal cortex, as well as in insular and ventral striatum function. Disturbances in the regulation of negative emotions also appear to be significant, and anger, fear, or sadness can often precede a binge-eating attack. Therefore, in addition to pharmacotherapy, psychotherapy plays a crucial role in the treatment of BED [55].

### 2.6. The CNS—Site of Action of Anti-Obesity Medications

Increasing knowledge of the pathomechanisms underlying obesity has led to the discovery of new strategies for its treatment. However, not all of these discoveries have been applied, due to unsatisfactory safety profiles or effectiveness; therefore, work on obtaining new substances is still ongoing, and their target points of action are various tissues and systems, including the CNS.

#### 2.6.1. Centrally Acting Drugs Currently Approved by the FDA

Phentermine belongs to the group of sympathomimetics and acts centrally by increasing the release of serotonin, norepinephrine, and dopamine, thereby reducing appetite [56]. Due to its lower dopamine-releasing potential compared to other representatives of this group, its use carries a lower risk of addiction. Phentermine is intended for short-term use (less than 12 weeks), with available doses ranging from 15 to 37.5 mg/day, and is administered orally as a single dose or in two divided doses [57]. In a 36-week randomized controlled trial (RCT), a decrease of up to 8.2 kg in placebo-subtracted mean weight was recorded. The most common side effects reported during use were sleep disorders, dizziness, dry mouth, weakness, constipation, and heart palpitations [56]. Administration of phentermine with topiramate, which is a GABA agonist that additionally increases phentermine’s effectiveness, has been approved for long-term use in weight management [58]. In a 1-year RCT, placebo-subtracted weight loss ranged between 8.6 and 9.3% in patients using 15 mg of phentermine and 92 mg of topiramate, which is the maximum combined dose [56,59].

Another centrally acting compound—bupropion—is a dopaminergic and noradrenergic agent that acts on the arcuate nucleus and causes weight loss by increasing satiety and energy expenditure. It stimulates the secretion of POMC, which, in turn, leads to the release of α-melanocyte-stimulating hormone (α-MSH), which affects the MC4R. The addition of naltrexone improves the secretion of POMC by influencing its μ-opioid receptor and inhibiting the autoinhibitory effect of β-endorphin secreted by POMC [56,60]. In the COR study, patients receiving a dose of 32 mg of naltrexone along with 360 mg of bupropion achieved a placebo-subtracted weight loss of 4.8%, while in the other COR studies the results differed, achieving as much as 9.3% weight reduction [61]. The most common reported side effects occurring after this combination were nausea, dizziness, sleep disturbances, and constipation [56].

GLP-1 receptor agonists (GLP-1RAs) such as liraglutide and semaglutide, initially used in the treatment of T2DM, are currently approved by the FDA for weight management and have been gaining an increasingly strong position on the anti-obesity drug market. Due to the presence of GLP-1 receptors in various tissues and organs, they also have multidirectional effects [62,63]. GLP-1 is secreted, among others, from the brain’s preproglucagon neurons in the nucleus tractus solitarii and works within the paraventricular nucleus and the arcuate nucleus, causing satiety [64]. Moreover, it turned out that GLP-1 increases thermogenesis in brown adipose tissue (BAT) by activating neurons in the dorsomedial hypothalamus and ventromedial nucleus. Progressive weight loss may also be related to the effects on the reward system and dopamine release, but the mechanisms have not been sufficiently researched [65]. Numerous clinical trials have documented that liraglutide and semaglutide therapies lead to weight loss compared to placebo. As for liraglutide, at a 3 mg daily dose along with lifestyle modifications, in the SCALE Obesity and Prediabetes trial, it showed weight reduction at the level of 8.0 (6.7)% in the group with obesity vs. 2.6 (5.7)% in the placebo group at 56 weeks [66,67]. The effectiveness and safety of semaglutide at 2.4 mg weekly were studied in the Semaglutide Treatment Effect in People with Obesity (STEP) trials for 68 weeks. For example, in the STEP 1 study, a 14.89% reduction in body weight was recorded in the group with obesity, compared to 2.49% in the placebo group.

Tirzepatide, a new molecule recently approved by the FDA for the treatment of obesity, is a dual receptor agonist acting on the previously mentioned GLP-1 receptors and glucose-dependent insulinotropic peptide (GIP) receptors. It turned out to be more effective in reducing HbA1c levels and, above all, in reducing body weight compared to GLP-1R agonists, as proven in the SURPASS and SURMOUNT studies [68,69,70,71,72]. The effect on the GIP receptor seems to be responsible for the increased effectiveness. In animal models, it interacts synergistically with GLP-1, enhancing the anorectic effect in the CNS. Additionally, an increased expression of POMC was also demonstrated [73]. Activation of the GIP receptor in the hindbrain has another positive effect, which is the reduction in the emetogenic effect listed as one of the most common side effects of GLP-1RAs. However, it seems that in the case of tirzepatide, side effects from the GI tract cannot be completely avoided due to the strong GLP-1 component [65].

The research on potential new incretin drugs is not limited only to dual agonists, but the subjects of interest also include multiagonists such as retatrutide (LY3437943), which is an agonist of the GLP-1, GIP, and glucagon receptors. There are other triple combinations that have been investigated as well, such as GLP-1, oxyntomodulin, and PYY receptor agonists [74,75].

For patients with monogenic syndromes of obesity, the FDA has approved two drugs. The first one, setmelanotide, was approved in 2020, and it is used in patients with POMC, PCSK1, or leptin receptor (LEPR) deficiencies, which result in insufficient activation of the MC4R and, consequently, cause excessive appetite and severe obesity developing in early childhood. Administered subcutaneously, it has a stimulatory effect on the MC4R and reduces hunger, decreases caloric intake, and increases energy expenditure in animal models [67,76,77]. Setmelanotide is a relatively well-tolerated drug, and the most frequently observed side effects were injection site reactions and hyperpigmentation [78]. During a study on 10 participants with POMC and 11 with LEPR deficiency, 80% in the first group and 45% in the second achieved at least 10% weight loss at approximately 1 year, and reduction in hunger was observed at 27.1% (*n* = 7; 90% CI −40.6 to −15.0; *p* = 0.0005) in the POMC trial and 43.7% (n = 7; 90% CI −54.8 to −29.1; *p* < 0.0001) in the LEPR trial [78].

Metreleptin, being a leptin analogue administered once-daily subcutaneously, is used in the treatment of obesity in patients with congenital leptin deficiency or congenital/acquired lipodystrophy. Its mechanism of action is based on POMC neuronal stimulation and NPY neuronal inhibition, thereby increasing satiety. A prospective non-randomized study with 17 patients noticed a reduction in lean body mass reaching 2.0 kg (*p* = 0.005) at 6 months compared to baseline [67,79], although the FDA has not yet approved any leptin analogues for the treatment of non-monogenic obesity [56].

#### 2.6.2. New Possibilities in Obesity Management

Extensive investigations have proven the key role of the CNS in regulating appetite and satiety. The consequence of these conclusions was the initiation of a search for new centrally acting molecules that could be used in the treatment of obesity. This paragraph highlights several examples of the molecules remaining in the center of scientific interest as new potential drugs for obesity.

Tesofensine (also known as NS-2330) acts as a noradrenaline, serotonin, and dopamine reuptake inhibitor, thus causing appetite suppression and subsequent weight loss. In four randomized, double-blind, multicenter trials in patients with Alzheimer’s and Parkinson’s disease, tesofensine led to a 4.0% weight loss without any intentional diet or lifestyle changes [80]. In the phase 2 trial, 203 obese patients were administered tesofensine in 0.25, 0.5, and 1 mg doses, and the observed weight loss reached 4.5, 9.1, and 10.6 kg, respectively. An additional advantage of the drug was the reduction in HbA1c, insulin, total cholesterol, and triglyceride levels. The side effects include an increase in blood pressure and heart rate at the highest dose of tesofensine, which may be related to its sympathetic effect [81,82]. In the 24-week randomized, double-blind phase 3 Viking study, with 372 participants receiving 0.25 mg/0.5 mg of tesofensine or placebo, a significant and superior weight loss (average: 10%) was achieved in both tested populations, and tesofensine was well tolerated, with few side effects reported [83]. Interestingly, tesofensine in combination with metoprolol remains the subject of research for possible use in patients with obesity caused by hypothalamic injury (NCT03845075) or Prader–Willi syndrome (NCT03149445) [56].

Oxytocin produced by the hypothalamus is involved in the body’s metabolic [84] regulation, control of energy expenditure, and eating behavior, especially the intake of carbohydrate foods. In animal models, its deficiency leads to obesity [85]. The potential anorexigenic effect is currently the subject of several studies in a group of patients with hypothalamic obesity and excessive appetite in Prader–Willi syndrome; however, the results are inconclusive. In a randomized, double-blind, placebo-controlled trial, 61 obese adults were divided into two groups—the first receiving intranasal oxytocin at a dose of 24 IU four times a day, and the second receiving a placebo for 8 weeks. Interestingly, there was no difference in body weight change between the groups (0.20 kg vs. 0.26 kg; *p* = 0.934), and no beneficial effect of oxytocin on resting energy expenditure or body composition has been described [86,87].

NPY antagonists are another group of drugs that are being investigated for possible use in the treatment of obesity. By antagonizing the orexigenic effect of NPY in the hypothalamus, they stop the activation of NPY receptor type 5 (NPY5R) and, thus, the promotion of food intake [88]. In a phase 2a trial with 342 obese patients receiving 400 mg or 1600 mg of velneperit (an NPY5R antagonist) once a day, dose-dependent efficacy was achieved, with good drug tolerability—in the group with the 1600 mg dose, the average weight loss was 5.3 kg (5.6%) vs. 2.5 kg (2.7%) in the group with 400 mg [56,89]. These satisfying results were not repeated in the phase 2b trial, where velneperit (400 mg dose) was tested as monotherapy and in combination with orlistat (120 mg) in 486 participants [40,75].

Cagrilintide (previously known as NNC0174-0833) is a long-acting amylin analogue that, in combination with semaglutide, remains an object of interest among researchers, appearing to have an additive effect on appetite reduction. Cagrilintide resembles amylin, which is naturally produced by pancreatic B cells in response to food supply and, thus, inhibits appetite by influencing the brain regions responsible for satiety in the homeostatic and hedonistic mechanisms. Additionally, it delays gastric emptying and reduces glucagon secretion [90,91]. In a single-center, placebo-controlled, multiple-ascending-dose phase 1b trial, patients were administered cagrilintide subcutaneously at a gradually increasing dose (0.16, 0.3, 0.6, 1.2, 2.4, and 4.5 mg) once a week, along with 2.4 mg of semaglutide (also once a week), or 2.4 mg of semaglutide with placebo. At 20 weeks, greater weight loss was observed in the cagrilintide plus semaglutide group—15.7% for 1.2 mg of cagrilintide, and 17.1% for 2.4 mg of cagrilintide, while only 9.8% for semaglutide with placebo. The most common side effects concerned injection site reactions, GI symptoms (vomiting, diarrhea, constipation, nausea), headache, and nasopharyngitis [92,93].

Another group that could potentially be used in the future to treat obesity is the cannabinoid-1 receptor antagonists. Their action is based on the influence on two types of cannabinoid receptors—CB1R and CB2R. The former occur mainly in various areas of the brain, including the cerebral cortex, cerebellum, basal ganglia, and hippocampus, and their stimulation causes hyperphagia [94]. CB1R antagonists suppress appetite and, thus, influence body weight loss [95]. The first representative of the CB1R antagonists approved for use was rimonabant, which, in addition to a significant decrease in body weight, had a beneficial effect on the lipid profile and glycemia. However, due to the serious side effects observed during its use (psychiatric disorders including mood disorders and suicidal thoughts), the drug was withdrawn from the market [96]. Appetite-reducing effects and improved metabolic profile in patients were also observed with another CB1R antagonist, namely, AM251 and AM4113 molecules, which may act both centrally and peripherally, with a lower risk of adverse psychiatric events [56,97,98].

Recently, ghrelin O-acyltransferase inhibitors have become a topic of studies in metabolic diseases [99]. These compounds prevent octanoylation on a specific serine side chain in ghrelin, preventing the activation of growth hormone secretagogue receptor 1a (GHS-R1a). Initial studies with the compound BI 1,356,225 show its acceptable tolerability, but the weight-reducing potency of these drugs seems disappointing [100]. Nevertheless, we are eagerly awaiting the further development of medications acting on this pathway, due to its potential hunger-mitigating properties.

## 3. Gastrointestinal (GI) Tract

Taking into consideration the role of the GI tract in terms of obesity, it is necessary to evaluate it from several points of view. Naturally, it is responsible for the uptake of nutrients. However, underlying mechanisms such as dysregulation of motility and altered gut microbiome composition should also be covered.

### 3.1. Diminished Absorption

Bariatric surgery remains a vital option in the treatment of obesity, especially its advanced stages. Its efficacy, due to restrictive or bypass procedures, is high. Nevertheless, it is generally non-reversible and sometimes leads to malabsorption and other surgical complications (including fatal outcomes). A pharmacological approach to affect digestion and ingestion is currently based on lipase inhibition using orlistat. Reducing triglyceride’s enzymatic degradation leads to a significant reduction in caloric intake and results in a weight loss reaching 5% [101]. Unfortunately, side effects limit the extensive use of the drug. Nevertheless, a study on combined therapy affecting lipid and carbohydrate ingestion with orlistat and acarbose has been conducted, showing some promising results [102].

Another interesting approach that is currently explored is to mimic “bypass surgery” using polymer coating of the intestinal mucosa, which results in reduced food absorption. GLY-200 is a polymer that is used in phase 2 clinical trials and seems effective and well tolerated [103].

### 3.2. Motility

The motility throughout the entire alimentary tract may be changed in obese patients, and the gut–brain axis and GI signaling hormones play a crucial part in the pathophysiology of motility-related disorders [104]. Some of them seem to be promising targets in weight management.

#### 3.2.1. GI Signaling Hormones

The GI neuroendocrine peptides that are produced and released by specialized cells include ghrelin, gastrin, pancreatic polypeptide (PP), peptide YY (PYY), cholecystokinin (CCK), somatostatin (SST), glucagon, glucose-dependent insulinotropic peptide (GIP), glucagon-like peptide 1 (GLP-1), glucagon-like peptide 2 (GLP-2), and vasoactive intestinal peptide (VIP), among others [105].

##### Ghrelin

This “hunger” hormone is secreted while fasting, mainly by mucosal cells located in the stomach and, to a lesser extent, in the duodenum. In its structure and function it resembles motilin, promoting gastric motility [106]. In obese patients, this prokinetic effect is limited due to decreased ghrelin levels, resulting in delayed gastric emptying, inhibited pyloric relaxation, and reduced intestinal motility [107,108].

##### Gastrin

Gastrin is secreted by G cells located in the stomach and duodenum in response to food intake. In addition to stimulating gastric acid secretion, this hormone also plays a role in gallbladder emptying and increases GI motility [109].

##### Pancreatic Polypeptide (PP)

Pancreatic polypeptide is a member of the neuropeptide Y family with a structure homologous to that of NPY and PYY. It is secreted after meals during digestion, mainly by pancreatic F cells, and reduces gastric emptying [110]. Interestingly, in obese patients, postprandial PP levels are low, which leads to the inhibition of intestinal motility [111].

##### Peptide YY (PYY)

Peptide YY is produced and secreted postprandially by small- and large-intestine endocrine cells, contributing to the inhibition of hydrochloric acid secretion and contraction of the gallbladder. Additionally, in obese patients, increased PYY levels, as a negative regulator of ghrelin release, cause GI transit time prolongation [108].

##### Cholecystokinin (CCK)

Cholecystokinin is secreted postprandially in the small intestine, especially by duodenal L cells, and physiologically stimulates secretion of pancreatic hormones and bile, but it also affects gastric emptying [112]. In obese patients, postprandial CCK levels are elevated, and as a result of negative regulation of ghrelin release and via acting on intestinal CCK receptors, it causes delays in gastric emptying and inhibits intestinal motility [108].

##### Somatostatin (SST)

Somatostatin is mainly secreted centrally by hypothalamic neurons and peripherally by D cells in the pancreas, as well as in smaller amounts by other tissues. In patients suffering from obesity, increased SST levels, being a negative regulator of both gastrin and ghrelin’s release, affect the GI tract through inhibition of gastric motility [108,113].

##### Glucagon

Glucagon is the main secretory hormone produced by A cells in the pancreas, which are mostly stimulated during hypoglycemia. In the liver, glucagon leads to hepatic glucose production, whilst centrally mediated glucagon receptor activation may cause appetite and food intake to decrease. Apart from that, it has an inhibitory effect on GI motility and may slow gastric emptying [114]. Importantly, fasting hyperglucagonemia may be present in obese individuals irrespective of diagnosed T2DM, and it might be connected with possible hepatic glucagon resistance due to steatosis [115,116].

##### Glucose-Dependent Insulinotropic Peptide (GIP)

Glucose-dependent insulinotropic peptide is the incretin hormone secreted by K cells in the small intestinal mucosa in response to chronic high fat consumption, and it is elevated in obese nondiabetic subjects. In addition to enhancing postprandial insulin secretion (the so-called “incretin effect” occurring when glucose is taken orally) and its impact on appetite suppression, GIP also negatively regulates ghrelin release and inhibits gastric emptying [108,117].

##### Glucagon-like Peptide 1 (GLP-1)

Glucagon-like peptide 1 is another example of an incretin hormone, which is secreted peripherally by intestinal L cells and by A cells in the pancreas, but also by the CNS. Although chronic fat intake promotes GLP-1 production [118], some data suggest that increased GLP-1 secretion is driven by the greater percentage of excess caloric intake [117]. GLP-1, similarly to GIP, enhances insulin secretion after a meal and suppresses appetite [117], but as another negative regulator of ghrelin release it also affects GI motility, leading to delayed gastric emptying and inhibiting intestinal motility [108]. This reduction in the gastric emptying rate powerfully decreases the rate of entry of nutrients into the bloodstream in patients with obesity, which may be beneficial in weight reduction [119].

##### Glucagon-like Peptide 2 (GLP-2)

Glucagon-like peptide 2, by analogy with GLP-1, is secreted by intestinal L cells as a response to the intake of food, and it binds to its receptors in the gastric fundus, resulting in postprandial gastric relaxation. Furthermore, elevated GLP-2 levels in obese people may also inhibit bowel movements [108,120].

##### Vasoactive Intestinal Peptide (VIP)

Vasoactive intestinal peptide is a neuroendocrine peptide hormone produced in both central and peripheral neurons. The main localizations of VIP are myenteric and submucosal neurons and nerve endings in the GI system [121,122]. In addition to the vasodilatory effect, VIP may also induce a vast number of effects in the GI tract through its receptors, including secretional regulation, control of pancreatic enzymes’ release, and gut contractility modulation. For example, the stomach’s smooth muscles are relaxed due to VIP activation [123,124]. Such a relaxant response of GI smooth muscles may be affected by a high-fat diet, which modifies nitric oxide secretion and, thus, results in enhanced VIP release in the myenteric plexus. This is why increased levels of VIP cause intestinal motility inhibition in obese subjects [108].

##### Amylin

As mentioned earlier, amylin is produced by pancreatic B cells and co-secreted with insulin in response to nutrient intake. It is primarily involved in the regulation of glucose levels by inhibiting glucagon secretion [125]. After being secreted, amylin activates its specific G-protein-coupled receptors in different tissues. In addition to the satiety-modulating function in the CNS, which seems to be dependent on a direct action on amylin receptors in the area postrema, it also affects GI tract motility, presumably by vagus nerve signaling, causing a delay in gastric emptying without influencing small intestinal or colonic transit [126]. A reduction in food intake does not result in a reduction in energy expenditure [127]. Plasma levels of amylin are increased in obese subjects, and in animal models chronic infusion of amylin into the brain decreases body weight gain and adiposity [128].

##### Oxyntomodulin

Oxyntomodulin is a peptide that is co-secreted with GLP-1 and glicentin from the intestinal L cells as a consequence of nutrient intake. Structurally, it is a derivative of proglucagon, which is produced and released by the endocrine gut cells after enzymatic processing by the precursor prohormone convertase 1/3 [129]. Being a dual agonist of GLP-1 and glucagon receptors, it combines the effects of both of these enterohormones [130]. In the GI tract, it decreases the secretion of gastric acid and pancreatic enzymes, whereas intravenous infusion of synthetic oxyntomodulin (proglucagon 33–69) in humans results in decreased gastric emptying and inhibits postprandial gastroduodenal motility [131]. Oxyntomodulin secretion in morbid conditions has not been specifically and widely investigated, but the available data suggest that the meal-induced responses were slightly decreased in obese subjects and in subjects with T2DM [129,132].

##### Leptin and NPY

Leptin, as a hunger-sensing hormone, is secreted by adipose tissue and the gastric mucosa, and its plasma concentration correlates with elevated BMI [133]. In obese patients, the higher the level of leptin, the higher the saturation of its receptors in the hypothalamus. Developed leptin resistance leads to dysregulation of satiety by inhibition of postprandial appetite suppression and promotes energy balance disturbances. Other consequences of hyperleptinemia in obesity are connected with the GI tract and include decreased esophageal motility [134]. Moreover, in animal models, exogenous leptin administration delayed gastric emptying and decreased small intestinal motility [135].

NPY, as mentioned before, is a neurotransmitter secreted by visceral fat tissue and is elevated in obese people. It stimulates appetite, contributing to body weight gain. NPY acts on its gastric receptors, which leads to a delay in stomach emptying. Additionally, along with VIP and nitric oxide, it inhibits colonic secretion and motility [108,136].

#### 3.2.2. Current Anti-Obesity Therapeutics Affecting GI Motility

Certain molecules presented above have already been utilized as targets for current anti-obesity drugs, and some others do have potential to be exploited as possible pathways towards the development of novel therapeutics.

The GI tract itself constitutes the site of action for several weight-loss medications, and even though they engage specific mechanisms involving GI motility, the impact of these drugs is usually multidirectional and not limited to the digestive system.

As an example, GLP-1RAs such as Saxenda^®^ (liraglutide) administered daily and Wegovy^®^ (semaglutide) administered once a week, apart from the abovementioned central effect on decreasing appetite, act peripherally as well. These injectable anti-obesity therapeutics mimic the effects of native GLP-1. They target the pancreas, which contributes to the increase in insulin secretion, but they also influence the GI tract by delaying gastric emptying and decreasing gut motility [137]. This slowdown in stomach emptying meaningfully reduces the rate of entry of nutrients into the bloodstream in patients with obesity, which is one of the possible mechanisms contributing to weight reduction in obese patients.

It is worth mentioning another GLP-1RA, dulaglutide, which is an FDA-approved drug for the treatment of T2DM and contributes to the reduction in cardiovascular mortality in diabetic patients. In the AWARD clinical trial, the efficacy and safety of different weekly dulaglutide doses were examined in patients with T2DM inadequately controlled with metformin. At 36 weeks, escalation from 1.5 mg to 3.0 mg or 4.5 mg of dulaglutide provided clinically relevant, dose-related reductions not only in HbA1C concentration, but also in body weight, with a similar safety profile [138]. Even though dulaglutide is not officially registered for weight management, for the above reason it is sometimes prescribed off-label as an anti-obesity medication [139].

As for the dual incretin receptor agonists, at the moment there is only one representative on the market, namely, tirzepatide. This new co-agonist of both GLP-1 and GIP receptors is currently available in injectable form under the trade names Zepbound^®^ [140] and Mounjaro^®^ [141]. In addition to the known effects of GLP-1 receptor activation, tirzepatide, by resembling native GIP, has an additional influence on gastric emptying delay. This is one of the reasons why, in comparison to the GLP-1Ras, this dual agonism entails the enhancement of effectiveness, which in obese subjects results in greater weight reductions [142].

**Figure 3:**
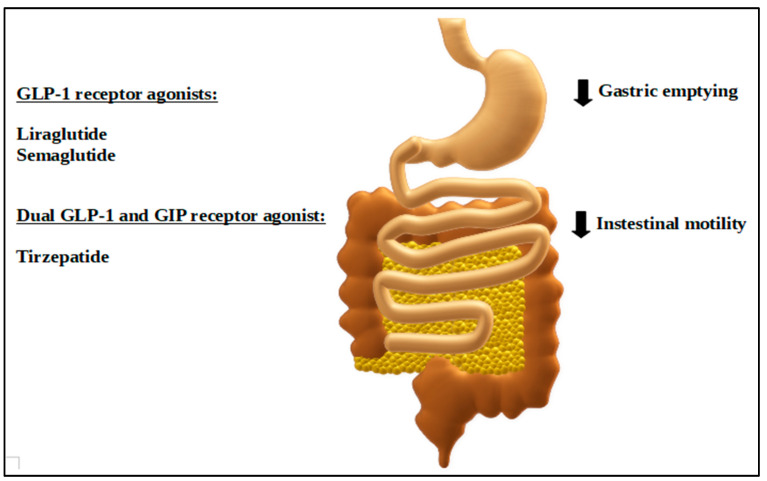
Liraglutide, semaglutide, and tirzepatide delay gastric emptying and decrease intestinal motility. The abbreviations are defined as follows: GIP—glucose-dependent insulinotropic polypeptide; GLP-1—glucagon-like peptide 1.

The impact of liraglutide, semaglutide, and tirzepatide on GI motility is graphically depicted in Figure 3, while the currently registered drugs for body weight management, including those affecting the GI tract, are summarized in Table 1.

#### 3.2.3. Potential Novel Anti-Obesity Molecules Influencing GI Motility

The myriad potential targets described in previous paragraphs are currently being intensely explored. There are many studies on obesity management with respect to potential new therapeutic compounds, which may possibly affect GI motility. Most of them, including incretin-based agents, in addition to their established effects on central appetite control, act on gastric emptying, contributing to satiation and, thus, resulting in weight loss.

##### Oral GLP-1 Receptor Analogues

Interestingly, on the market there is the well-known semaglutide, yet it is also available in an oral formulation, which is currently approved for the treatment of T2DM in a once-daily dosage and is being evaluated by regulatory authorities for obesity [149]. In Rybelsus^®^, the formulation of semaglutide tablets was possible due to the utilization of a specific delivery method based on permeation enhancers, i.e., the substances added to peptide-based drugs intended for oral administration in order to facilitate their proper penetration through the GI epithelium. In this particular case, semaglutide was successfully co-formulated with salcaprozate sodium (SNAC) [150]. Although the circulating levels of the active substance and biological efficacy were comparable to that of the subcutaneous drug form [149], it seems that higher daily doses, such as even 50 mg of oral semaglutide, would be more appropriate in order to meaningfully decrease body weight in adults with overweight or obesity but without T2DM [151].

It is speculated that, in the near future, there will be available another oral GLP-1RA for the treatment of obesity. Danuglipron is an oral small-molecule GLP-1RA with proportionate efficacy to that of injectable peptide analogues in a humanized model. In patients with T2DM, a significant weight loss at 16 weeks was noted [149,152]. Furthermore, orforglipron, an oral non-peptide GLP-1RA, is expected to be administered without the needed interval between drug intake and meal consumption. Daily doses of orforglipron within the range of 12–45 mg were associated with weight loss of over 10% at 36 weeks in obese patients without T2DM [149,153].

##### Cagrilintide—A Novel Amylin Receptor Analogue

Pramlintide, the first short-acting amylin analogue, was approved for the treatment of T2DM in 2005 and is used as an adjunct to insulin. Although it showed significant body weight reduction additionally to a glucose control in clinical trials for T2DM, it is not approved for the treatment of obesity [92]. Another therapeutic prospect is cagrilintide, a novel long-acting amylin receptor agonist that, administered subcutaneously, increases satiety signals centrally and decreases gastric emptying peripherally [92]. Combined with semaglutide, cagrilintide appears to have an additive effect on appetite reduction. This combination therapy, known as CagriSema, is currently being evaluated in the REDEFINE 2 study (phase 3), in which cagrilintide (2.4 mg) and semaglutide (2.4 mg) are administered subcutaneously once a week in participants with overweight or obesity and T2DM. The primary outcome measure of this trial is the relative change in body weight from baseline to week 68, and the main secondary outcome measure is achievement of at least 20% weight reduction. The study completion date is estimated at 29 January 2025 [154].

##### Novel Dual Incretin-Based Analogues

Another group of twincretins constitute co-agonists of GLP-1 and glucagon receptors, with the following dual analogues being currently under investigation: survodutide (BI 456906), mazdutide (IBI362 or LY3305677), and cotadutide (MEDI0382).

In a preclinical study in obese, insulin-resistant mice, survodutide, due to its dual agonism, demonstrated greater body weight reductions compared with maximally effective doses of semaglutide. This was achieved by the inhibition of gastric emptying, along with food intake suppression and energy expenditure increase, while maintaining normoglycemia. The efficacy of the delay in gastric emptying was similar to that of semaglutide, but with an approximately 10- to 20-fold lower potency at the GLP-1 receptor, which suggests that plasma protein binding is markedly higher for survodutide in mouse plasma [155]. More importantly, in a randomized, double-blind, placebo-controlled, dose-finding phase 2 trial, survodutide administered subcutaneously (0.6, 2.4, 3.6, or 4.8 mg) once a week for 46 weeks (20 weeks of dose escalation and 26 weeks of dose maintenance) in adults with a BMI of at least 27.0 kg/m^2^, without diabetes, significantly decreased body weight—over half of the participants receiving a 4.8 mg dose reached at least 15% reduction in body weight [156]. Consequently, survodutide seems to be a promising novel anti-obesity medication, which is expected to be confirmed in the next phase of trials.

As far as mazdutide and cotadutide are concerned, Bixin Deng et al. assessed their safety and efficacy in the first such systematic review and meta-analysis. They investigated 14 RCTs (phases 1 and 2), of which all were placebo-controlled trials and 5 concerned mazdutide, whereas 9 utilized cotadutide. In total, 1256 patients with T2DM, obesity, or both were analyzed, and compared to the placebo these molecules significantly contributed to weight loss and improved control of glycemia, offering another potential tool in the fight against obesity [157].

Under research, there is also pemvidutide (ALT-801), another GLP-1/glucagon receptor dual agonist, which has been tested primarily in a mouse model of metabolic dysfunction-associated steatohepatitis (MASH) [149]. In addition to improving liver steatosis, it caused significant weight loss (approximately 25%) in overweight and obese patients without diabetes. These data support the development of pemvidutide as a promising treatment for obesity, with a hypothesized minimization of GI intolerance [158].

##### Maridebart Cafraglutide (AMG-133)—A Novel GLP-1 Receptor Agonist and GIP Receptor Antagonist

Contrary to the GLP-1/GIP co-agonists, maridebart cafraglutide (AMG-133) is a GLP-1 receptor agonist and GIP receptor antagonist. This bispecific molecule is engineered by conjugating a fully human monoclonal anti-human GIP receptor antagonist antibody to two GLP-1 analogue agonist peptides [159]. Apart from its GLP-1 receptor agonism, the GIP receptor antagonism promotes decrease in body weight due to reduction in food intake and improved fat cell metabolism [160]. Promising preclinical tests in obese animals have been supported by phase 1 clinical trial results in humans with obesity, which confirmed the potency of maridebart cafraglutide (AMG 133) for weight loss without severe adverse GI effects [161]. Maridebart cafraglutide (AMG-133) is currently being evaluated in adult patients with overweight or obesity, with or without T2DM, in a phase 2 clinical trial [162].

##### Novel Triple Incretin-Based Analogues

At present, there are ongoing clinical trials regarding triple incretin-based co-agonists. Retatrutide (LY3437943), being a GLP-1, GIP, and glucagon receptors agonist, has been the first one to enter clinical trials for the treatment of T2DM and obesity [163]. In comparison to native hormones, it displays significantly higher potency at the human GIP receptor, with reduced potency at the human GLP-1 and glucagon receptors [74]. Despite this, retatrutide still affects the GI tract and causes a delay in gastric emptying. In one of the clinical studies, the greatest effect on gastric emptying developed after the first dose, and the maximum extent of delayed stomach emptying was similar to that previously noted with tirzepatide [164]. Having a triple mechanism of action, retatrutide, at a subcutaneous dose of 12 mg once weekly, caused up to almost 17% body weight loss in obese patients, while maintaining a safety profile comparable to that of both GLP-1RAs and GIP/GLP-1 receptor co-agonists [165,166].

Another GLP-1/GIP/glucagon receptor triagonist, efocipegtrutide (HM15211), is undergoing preclinical studies for its efficacy in the treatment of T2DM, MASH, and Parkinson’s disease [167]. Although it has not yet been investigated for obesity management, in mice models, in addition to a reduction in hyperglycemia, there was significant weight loss and increased energy expenditure in comparison to liraglutide [168].

It is worth mentioning the next such polyagonistic approach with a combined impact on GLP-1, oxyntomodulin, and PYY receptors. In a randomized, single-blind, placebo-controlled study, Preeshila Behary et al. subcutaneously infused GLP-1, oxyntomodulin, and PYY for 4 weeks in 24 subjects with obesity and prediabetes/T2DM. This tripeptide hormone infusion suggested a significant reduction in restraint eating in addition to metabolomic and glycemic effects [169], but further randomized, controlled trials with these promising agents are needed [149].

A new GLP-1/glucagon/gastrin receptor triagonist compound has been studied in diabetic and obese mice. It showed superior glycemic control compared to cotadutide (MEDI0382) and liraglutide, with improved weight loss effects as compared with liraglutide, but similar to those of cotadutide (MEDI0382). It is a possible therapeutic target and future treatment option in obesity and T2DM [167,170].

#### 3.2.4. Adverse GI Motility Events Related to Anti-Obesity Therapy

GI disorders are the most common treatment-emergent adverse events of the discussed incretin-based drugs, which is in line with what would be expected based on their mechanisms of action. Patients may report diarrhea, constipation, nausea, decreased appetite, vomiting, dyspepsia, or abdominal pain, and in most cases the severity is generally mild-to-moderate and temporary, depending on the dose [171]. However, the desired effect of delayed gastric emptying may in some cases result in gastroparesis [172]. Such a drug-induced, morbidly prolonged food retention in the stomach is currently in the area of interest of both anesthesiologists and endoscopists, because it may cause an increased risk of aspiration pneumonia associated with GI endoscopies. This is why holding GLP-1RAs before an endoscopic or surgical procedure, if possible, should be always taken into account in order to decrease this risk [173].

Additionally, it should be noted that not all drugs acting in the GI tract primarily affect motility. Orlistat, for instance, inhibits both pancreatic and gastric lipases and, thus, decreases dietary fat absorption, which may result in steatorrhea [174].

### 3.3. Microbiome

The term gut microbiome refers to microorganisms such as bacteria, fungi, and protozoa that inhabit the digestive tract [175]. Contrary to the original view that children are born with a sterile digestive tract, there is some evidence that the human intestinal microbiota starts to form during intrauterine life and bacteria are present in the placenta [176], amniotic fluid [177], and umbilical cord [178]. Moreover, microorganisms were detected in the meconium of neonates [179,180]. The colonization continues postpartum and is influenced by the type of birth [181], breastfeeding, and diet [182]. In adults, there are many factors affecting the exact composition of the intestinal microbiota [183]. These factors are both modifiable—including lifestyle [184,185], diet [186,187,188], use of antibiotics [189,190], use of other drugs [191], and many others—and non-modifiable (genetics) [192].

Despite difficulties in defining a healthy microbiota [193], there is some evidence that it might be disturbed in some conditions [194], including obesity [195,196]. Firstly, a study on healthy and obese twins showed that the gut microbiome in obesity is less heterogeneous [196]. The next variable that might be correlated with abnormal body weight is the change in the relative abundance of bacterial phyla such as *Firmicutes* and *Bacteroidetes*, often expressed as the *Firmicutes*/*Bacteroidetes* (F/B) ratio. Although some studies on mice [197] and on humans [198,199] have shown that higher levels of *Firmicutes* and lower levels of *Bacteroidetes* are associated with obesity, other studies did not confirm that [200]. The presence of some families or even species of microbes might be beneficial in terms of metabolic health as well. There are studies showing *Christensenellaceae* [201,202,203] and *Akkermansia* [202] as having a positive impact on human metabolism. Other bacteria that are thought to be associated with normal BMI are *Methanobrevibacter smithii* and *Bifidobacterium animalis*. On the other hand, an unfavorable impact might be exerted by *Lactobacillus reuteri* [204].

#### 3.3.1. Influence of Microbiota on Body Weight

Bacteria inhabiting the digestive tract influence many bodily functions, especially metabolism and the host’s energy balance. This is done in many ways, ranging from the production of various substances via interactions with the immunological system to the influence on circadian rhythm. All of these mechanisms are interconnected [205].

##### Fermentation of Indigestible Carbohydrates

Not all substances delivered to the body with food can be absorbed in the small intestine. Such substances include starch, fiber, and some proteins [206]. They reach the colon, where microorganisms that are able to metabolize these particles are present. This process is called fermentation, and its main products include short-chain fatty acids (SCFAs) such as acetate, propionate, butyrate, and lactate, as well as gases—carbon dioxide, methane, and molecular hydrogen [207]. Then, other types of bacteria use gases in processes called methanogenesis, acetogenesis, and sulfate reduction [208]. Interestingly, SCFAs, despite their impact on receptors, serve as a source for gluconeogenesis (propionate) and lipogenesis (acetate) [206]. In this manner, depending on many details, including microbiome composition, organisms are able to obtain up to 10–30% of the energy needed for basal metabolism [209].

##### Short-Chain Fatty Acids (SCFAs) and Their Receptors

SCFAs, the end products of indigestible carbohydrates’ fermentation, are organic acids with one to six carbon atoms in a particle. The most common SCFAs in live organisms are acetic acid, propionic acid, and butyric acid, which are typically formed at an approximate molar ratio of 3:1:1, respectively, but may vary depending on many conditions. Only 5–10% of them are excreted with stool; the rest are absorbed in the colon [210,211,212]. Despite their energetic function discussed above, they interact with G-protein-coupled receptors (GPCRs) called free fatty acid receptor 2 (FFAR2, also known as GPR43) and FFAR3 (GPR41), which are present in various tissues. Other possible targets for SCFAs are hydrocarboxylic acid receptor 2 (HCAR2 or GPR109A) and GPR164 [211].

FFAR2s are mainly expressed in adipose tissue [213] and immunological cells, suggesting their role in immune response [214,215]. While their role in adipogenesis remains unclear, it is believed that their activation stimulates lipolysis [213].

In terms of FFAR3s, they are expressed, among others, in enteroendocrine cells, nerves, and pancreatic cells [216]. They are involved in energy regulation, and it has been shown that their activation might be beneficial in terms of maintaining normal body weight [217].

##### Bile Acids (BAs)

In the process of liver metabolism of cholesterol, primary BAs such as cholic acid and chenodeoxycholic acid (CDCA) are produced and conjugated with taurine or glycine and then secreted in bile. In the small intestine, it acts as a detergent, which increases the surface area of lipase action on ingested lipids. As far as the microbiome is concerned, it deconjugates primary BAs and transforms them into secondary BAs such as deoxycholic acid and lithocholic acid, which are excreted with feces or absorbed and transported back to the liver [218].

In addition to their digestive function, BAs are able to bind and activate nuclear receptors such as farnesoid X receptor (FXR), vitamin D receptor (VDR), pregnane X receptor (PXR), and GPCRs. In this manner, they may influence the immunological and barrier function of the gut [219].

Studies have shown that BA profiles differ in obese and normal-weight patients, suggesting that this might be one of the links connecting gut bacteria with metabolic diseases [220].

##### Gut–Brain Axis (GBA)

The concept of bidirectional connection of the CNS and GI tract is called the gut–brain axis. Many different and still not fully understood mechanisms take part in the mutual communication of these systems. They include neuronal, neuroendocrine, and immunological connections, which are greatly influenced by microbes that inhabit the intestines. GBA regulates many important bodily functions, particularly those associated with nutrition and metabolism [221].

Microbiota produce many substances, including neurotransmitters such as dopamine, noradrenaline, GABA, and tryptophan that may be engaged in interactions with the CNS. They are absorbed into the blood and transported to various parts of the body, including the brain and hypothalamus, where they exert their functions. However, it is probable that not all of them are able to cross the blood–brain barrier. Previously mentioned SCFAs might act in a similar manner as well [222].

Moreover, gut bacteria might alter the production of peptides by enteroendocrine cells in a direct or indirect way. There is evidence that the composition of the microbiota influences the synthesis of enterohormones such as GLP-1, CCK, and PYY, which are responsible for satiety [223,224].

##### Metabolic Endotoxemia

Lipopolysaccharide (LPS) is a component of the outermost membrane of Gram-negative bacteria, which can induce an immunological response after binding to Toll-like receptor 4 (TLR4) expressed on the cell surface of monocytes and other cells. This starts a pro-inflammatory cascade and leads to oxidative stress [225]. In contrast to septic shock, where LPS levels are greatly elevated and lead to multiorgan failure, in metabolic diseases LPS levels might be chronically elevated in smaller quantities, which can lead to deterioration of glucose tolerance and accumulation of adipose tissue [226,227]. Metabolic endotoxemia might be caused by increased gut permeability. The composition of the gut microbiota is a one factor influencing this parameter; for example, some bacteria might stimulate GLP-2 production, which contributes to sealing the intestinal barrier [228].

##### Energy Storage and Expenditure

It has been shown that the microbiota might influence the synthesis of fasting-induced adipose factor (FIAF), which is an inhibitor of lipoprotein lipase (LPL). The higher the activity of LPL, the higher the fat storage in adipose tissue. Moreover, gut bacteria affect energy expenditure by modulating the activation of AMP-activated protein kinase (AMPK), which is positively correlated with catabolic processes [229,230].

Another signaling pathway that can be altered by the microbiota is the endocannabinoid system [231]. Studies have revealed that its hyperactivity is associated with metabolic disorders such as excessive body mass, visceral obesity, and abnormal glucose metabolism [232].

##### Influence on the Internal Clock

Another possible, interesting mechanism linking gut commensals with metabolic disorders is their potential impact on the expression of genes associated with circadian rhythm in the liver. This impact might be exerted by metabolites produced by microorganisms [233]. Abnormalities in day–night rhythm caused by dysregulated expression of genes associated with it are a known factor contributing to metabolic disorders, including obesity [234].

#### 3.3.2. Potential Therapeutic Methods Primarily Affecting Microbiota

Potential therapeutic methods used in order to mitigate excessive body mass and its negative health consequences that affect the host’s microbiome include the modification of microflora composition with supplementation of prebiotics, probiotics, or synbiotics, or even with fecal microbiota transplantation (FMT) and supplementation of substances that are produced by commensal bacteria.

##### Prebiotics, Probiotics, and Synbiotics

Prebiotics are indigestible substances that stimulate the growth or action of defined species of bacteria in the host’s GI tract [235]. Probiotics are microorganisms supplied from the outside to treat diseases. Finally, synbiotics are formulations containing both prebiotics and probiotics [236].

In an umbrella review by Niloufar Rasaei et al., which took into account 97 meta-analyses on the influence of pre-, pro-, and synbiotics on anthropometric parameters such as BMI, body weight, and waist circumference in obese individuals, the authors showed a positive impact of these therapeutic methods in the general population. However, the authors emphasized that more well-designed studies are needed, especially those taking into account specific strains of bacteria [237]. Another meta-analysis including 26 RCTs showed beneficial effects of probiotics on body weight, body fat mass, and some known risk factors of cardiovascular diseases [238]. One more study summing up 20 RCTs suggested that probiotic supplementation might contribute to lowering BMI as well as hip and waist circumference [239].

Nicolás Farid Hamed Riveros et al., in their meta-analysis of 11 studies with 911 participants, revealed that *Bifidobacterium* genus probiotics reduce body fat mass and fat percentage without any effect on BMI and body weight [240].

In terms of *Lactobacillus*, it was shown that different species might have opposite effects on weight gain in humans [241]. However, meta-analysis of nine RCTs showed favorable effects associated with *Lactobacillus* supplementation on cholesterol concentration and fasting plasma glucose in obese patients [242].

With the development of technology used for the identification of microorganisms, the term next-generation probiotics (NGPs) was established, which refers to the microorganisms identified with novel technologies, and whose administration in the proper amount might be beneficial in some conditions [243]. In terms of obesity, *Akkermansia muciniphila* is believed to cause positive metabolic effects in the overweight—for example, reducing the waist-to-hip ratio or lowering fasting glucose [244]. One of the proposed mechanisms by which *A. municiphila* influences host health is its ability to degrade mucins in the gut [245]. Other promising NGPs include *Faecalibacterium prausnitzii*, *Eubacterium hallii*, *Bacteroides plebeius*, *Bacteroides uniformis*, *Hafnia alvei*, *Christensenella minuta*, and others [246].

Another intriguing option to affect the microbiota is to change the level of oxygen in the GI lumen using its chemical donors (XEN-101). Its development is in its early stages but, by using this novel approach, a successful modification of the microbiota might be achieved [247].

##### Fecal Microbiota Transplantation (FMT)

FMT is a procedure in which the microbiota of healthy donors is placed in the GI tract of people with a disease, with intention to treat it. The main disease cured with this method is infection caused by *Clostridioides difficile.* However, based on the previously mentioned facts, it could also be a promising therapeutic option for obese patients [248].

The meta-analysis performed by Proença et al., which took into account six RCTs and 154 patients, did not show significant differences in terms of anthropometric parameters 6 and 12 weeks after the procedure in recipients of lean donors’ microbiota in comparison with the placebo group. However, patients who underwent FMT had lower HbA1c concentrations and increased high-density lipoprotein (HDL) levels 6 weeks after the transplantation [249]. Another meta-analysis by Qiu et al., including nine studies and 303 participants, also did not indicate any positive impact of FMT on body weight, but it showed beneficial effects on HbA1c levels, insulin sensitivity, and HDL concentration [250]. One more meta-analysis of 10 RCTs (334 patients) by Zecheng et al. revealed that FMT recipients had lower caloric intake than patients from the placebo group, but these groups did not differ in terms of weight, BMI, or waist circumference. This study also indicated that FMT might improve parameters such as fasting glucose, lipogram, and blood pressure [251].

##### Sodium Butyrate

Sodium butyrate, as one of the SCFAs produced by the microbiota and exerting metabolic effects through G-protein-coupled receptors, is also a potential therapeutic agent in metabolic disorders. It has been tested mainly in animal models, showing that it may reduce body weight and, thus, prevent diet-induced obesity [252,253]. An RCT on 54 obese children showed that butyrate supplementation added to standard therapy improved BMI reduction, indicating that it might become a promising therapeutic method [254].

## 4. Energy Expenditure

### 4.1. Adipose Tissue

The human body contains two types of adipose tissue: white adipose tissue (WAT), and brown adipose tissue (BAT). BAT is specialized in energy expenditure through thermogenesis, while WAT is an energy store [255,256]. Fat is the largest energy reservoir in mammals. The most important tissues involved in fatty acid (FA) metabolism are skeletal muscle, adipose tissue, and the liver. In adipose tissue, FAs serve as an energy reserve for other tissues. In muscles, they are utilized to generate energy, while in the liver they are converted to triacylglycerols during the re-esterification process, which are secreted as very-low-density lipoproteins (VLDLs). Insulin stimulates fat storage and inhibits its mobilization in adipose tissue, which consequently leads to energy storage. It also inhibits VLDL secretion in the liver. Moreover, physical activity increases the utilization of FAs in muscles [257].

#### Bone Morphogenetic Proteins (BMPs) and Other Adipokines

White adipocyte differentiation is induced by some members of the bone morphogenetic protein (BMP) family. In contrast, BMP7 promotes the differentiation of brown preadipocytes even in the absence of other factors. It activates the brown adipogenesis program, including the induction of PRDM16 and PGC-1α. It also increases the expression of UCP1-, PPARγ-, C/EBPs-, Mapk14-, and PGC-1-dependent pathways. BMP7 induces the differentiation of mesenchymal progenitor cells towards BAT. The absence of BMP7 in embryos results in a significant deficiency of BAT and an almost complete absence of UCP1. In contrast, increasing BMP7 expression with adenoviruses leads to an increase in brown fat, increase in energy expenditure, and reduction in weight gain [256]. BMP7 belongs to a group of adipokines that includes adiponectin, leptin, retinol-binding protein 4 (RBP4), visfatin/nampt/PBEF, bone morphogenetic protein (BMP)-4, fibroblast growth factor 21 (FGF21), cathepsins, nesfatin-1, apelin, vaspin, progranulin, omentin, and lipocalin, among others [258]. Adipokines exert pleiotropic effects on other tissues, including glucose metabolism, immune response, myocardial contractility, vascular growth and function, adipogenesis and bone morphogenesis, blood pressure, cell adhesion, lipid accumulation in the liver, lipid metabolism, and other biological processes [259]. Their secretion is altered in adipose tissue dysfunction, making them candidates for the development of potential novel drugs for the future treatment of obesity [258].

### 4.2. Liver

The liver is the metabolic center of the human body, playing a pivotal role in the metabolism of carbohydrates, lipids, and proteins. It serves as a metabolic conduit between the skeletal muscles and adipose tissue. During periods of satiety, the liver converts glucose into glycogen and/or FAs, which then can be esterified in order to produce triacylglycerols, stored in hepatocytes, or secreted as VLDLs. During starvation or exercise, the liver provides energy substrates to tissues that require energy. The liver is responsible for the production of glucose through the process of gluconeogenesis, utilizing alanine and lactate produced by muscle and glycerol released from adipose tissue. Additionally, it produces ketone bodies in the process of β-oxidation, utilizing non-esterified FAs released from adipocytes [260].

### 4.3. Muscles

Despite accounting for 40% of body weight, skeletal muscles are responsible for 75% of insulin-stimulated glucose uptake, making them the main location for insulin-dependent glucose utilization [261]. The high requirement for ATP in the cross-bridge cycle makes muscle tissue a crucial component in maintaining the body’s energy homeostasis. Glycolysis is the main source of energy. The process commences with the transport of glucose across the plasma membrane by GLUT4, which is promoted by the activation of AMPK [262]. This serine/threonine kinase is activated in response to reduced muscle ATP concentrations, or pharmacologically by, for example, 5-aminoimidazole-4-carboxamide ribonucleoside (AICAR) and metformin, leading to insulin-independent glucose uptake [263]. The second source of energy is β-oxidation. FAs are transported into cells via various transporters, including FATP/SLC27A1, CD36, and FABP3. The primary ATP production pathway is mitochondrial oxidative phosphorylation (OXPHOS). The peroxisome proliferator-activated receptor γ (PPARγ) and its coactivator PPARγ 1α (PGC-1α) regulate aerobic metabolism through the regulation of gene expression, including MCAD, CPT1, LCAD, ACS, and FATP. AMPK exerts control over PGC-1α in two distinct ways: directly, through phosphorylation of PGC-1α, and indirectly, through the activation of SIRT-1 [262].

A multitude of endeavors are being undertaken to identify pharmacological agents that might substitute for exercise, which are known as exercise mimetics. The principal mode of action of these substances is based on the activation of AMPK. This kinase is directly activated by salicylate, AICAR, the small compound thienopyridine (A7769662), and benzimidazole derivatives (compound **911**), as well as indirectly (by promoting ATP or calcium accumulation) by glitazones, polyphenols (resveratrol), and biguanides such as metformin. Another promising substance is GW501516 (a selective PPARδ agonist), which has been shown to increase the expression of genes involved in FA metabolism and oxidative capacity. Efforts are also being made to influence adaptive thermogenic mechanisms. The primary source of this process is the futile circulation of Ca^2+^ via the sarcoplasmic/endoplasmic reticulum calcium ATPase (SERCA) pump. Additionally, a futile cycle involving simultaneous lipolysis and re-esterification of triacylglycerols represents another potential adaptive thermogenic mechanism. It has been demonstrated that eicosapentaenoic acid (EPA) can stimulate this process [264].

### 4.4. Compounds Affecting Energy Expenditure

#### 4.4.1. Conjugated Linoleic (CLA), Docosahexaenoic (DHA), and Eicosapentaenoic (EPA) Acids

In one study, supplementation with EPA/DHA or CLA increased oxygen consumption, carbon dioxide production, and energy expenditure in a mouse model. It is hypothesized that alterations at the mitochondrial level influence energy expenditure [265]. One human study using fish oil (FO) supplementation demonstrated an increase in resting metabolic rate by 14%, fat oxidation at rest by 19%, and lean body mass by 4% [266]. In another study, however, no effect was observed [267]. These two trials differed significantly in their study populations, with one comprising 24 females aged 66 ± 1 year and the other 26 males aged 22.8 ± 2.6 years. This may have influenced the results.

#### 4.4.2. β-Aminoisobutyric Acid (BAIBA)

This compound induces the transformation of white adipose cells into brown adipose cells, which contain thermogenin. The function of this protein is to facilitate the breakdown of lipids into carbon dioxide, water, and heat. Moreover, physical activity has been demonstrated to increase the transcription of peroxisome proliferator-activated receptor gamma coactivator 1-alpha (PGC-1α), a key player in mitochondrial function and biogenesis. The mediator of this metabolic process is BAIBA [268].

#### 4.4.3. Ginseng

A study with mice demonstrated that white ginseng has a beneficial effect on reducing body weight gain and white adipose tissue mass. The authors of the study suggested that this may be linked to the regulation of lipogenesis gene expression in WAT and delayed fat absorption. The group of mice that received Korean white ginseng extracts exhibited a reduction in the expression of lipogenesis-related genes: sterol-regulatory element-binding protein-1c (SREBP-1c), lipoprotein lipase (LPL), FA synthase (FAS), diacylglycerol acyltransferase 1 (DGAT1), and peroxisome proliferator-activated receptorγ2 (PPARγ2) [269]. Ginseng may also enhance energy utilization by stimulating the adenosine monophosphate-activated kinase pathway. Furthermore, it exerts an influence on food intake. The majority of studies conducted to date have been conducted on animal models. Further research is required on humans to ascertain the efficacy of ginseng in the treatment of obesity [270].

#### 4.4.4. Incretin-Based Drugs

Glucagon increases energy expenditure. This action is not contingent on the BAT [271]. The pleiotropic effect of GLP-1 analogues is well documented and has been extensively studied in numerous papers cited above. These analogues also affect peripheral lipid metabolism. In a study on liraglutide, this GLP-1 analogue was shown to reduce triglyceride and VLDL levels, calorie intake, and body weight, independent of diet. In lean rats, it increased the expression of the following genes/proteins in epididymal white adipose tissue and muscles: ChREBP, Acaca/ACC, Fasn/FAS, Scd1/SCD1, PPARα/γ, CPT1b, Cox4i1, and Ucp1/UCP1. These elements are involved in the processes of β-oxidation, thermogenesis, and lipogenesis, which suggest the use of FAs for energy expenditure. In contrast, in obese rats fed a high-fat diet, there was a reduction in the expression of phospho-ACC, ACOX1, and PPARγ/PPARγ in the liver, which suggests a recovery of lipid homeostasis. The muscles of obese rats also showed a reduction in the expression of PPARγ and the thermogenic factor UCP1 [272]. Furthermore, an increase in energy expenditure was observed for dual and triple agonists, with the triple GLP-1, GIP, and glucagon receptor analogue demonstrating superiority over the dual GLP-1 and GIP receptor analogue in this regard [74].

#### 4.4.5. Resveratrol and Epigallocatechin-3-Gallate

In an animal model using elderly, healthy mice, resveratrol was observed to reduce the respiratory quotient, increase FA degradation, and decrease FA synthesis. Resveratrol has been demonstrated to increase the expression and phosphorylation of AMPKα, which is an upstream inhibitor of ACC1. ACC1 plays a key role in FA synthesis [273]. In a study with humans investigating the effects of resveratrol in combination with epigallocatechin-3-gallate, no long-term effect of resveratrol on energy expenditure was demonstrated. Nevertheless, a reduction in visceral adipose tissue and in respiratory quotient was observed, both fasting and post-meal, along with an increase in the oxidative capacity of muscle fibers, with no effect on fat oxidation [274].

#### 4.4.6. R,S-1,3-Butanediol Diacetoacetate (BD-AcAc2)

A study with mice demonstrated that replacing carbohydrates with BD-AcAc2 in a high-fat diet (45% of overall kcal) resulted in a 26% reduction in mean weekly energy intake compared to the control group. Furthermore, the final body weight and fat mass were significantly lower in the BD-AcAc2 group compared to the pair-fed group, accompanied by an increase in resting and total energy expenditure after adjustment for lean body mass and fat mass. At the molecular level, it was observed that the aforementioned compound increased Ucp1, Dio2, and Pgc1a mRNA in brown adipose tissue compared to the pair-fed group. These markers are associated with mitochondrial uncoupling and thermogenesis. However, no differences in skeletal muscle mitochondrial oxidative capacity or coupling were observed between the groups [275].

#### 4.4.7. Methylphenidate

Methylphenidate, used to treat attention deficit hyperactivity disorder (ADHD) [276], increases both resting energy expenditure and postprandial energy expenditure in healthy men and women. At the same time, it does not affect the respiratory exchange ratio, heart rate, or systolic and diastolic blood pressure [277]. Furthermore, evidence indicates that it can reduce overall energy intake with a selective reduction in dietary fat [278]. Interestingly, in a group of children with ADHD, a reduction in energy expenditure was observed as a result of a reduction in physical activity [279]. In contrast, a reduction in energy intake from fat and carbohydrates was observed in a group of obese adolescents. This is likely due to the effect on the amount of dopamine in the brain [280].

#### 4.4.8. Other Substances

In addition to the previously discussed substances that affect the body’s energy expenditure, numerous others have also been investigated. A comprehensive discussion of those would exceed the scope of this study. These compounds include acetate [281], leptin (which was discussed in previous chapters), Chrysanthemum morifolium flower extract [282], angiopoietin-like 4 [283], celastrol [284], vanillic acid [285], and numerous others that are not listed above.

The other substances under ongoing clinical trials and with a potential impact on energy expenditure include angiotensin-(1–7) [286], mirabegron [287], salbutamol [288], ADI-PEG20 [289], empagliflozin [290], tadalafil [291], vericiguat [292], *Triticum aestivum* [293], LEAP-2 protein [294], maslinic acid [295], nicotinamide riboside [296], olfactory stimulation with distinct odors [297], raw whole almonds [298] and, last but not least, semaglutide [299].

## 5. Future Prospects

A vast number of studies are underway, involving substances that may play a crucial role in body weight management in the future. In Table 2, we summarize the currently pursued pharmacological interventions in obesity, with particular regard to their mechanisms of action, and including the clinical trial phase.

## 6. Conclusions

Obesity is currently the most common lifestyle-related risk factor for premature death, requiring effective treatment. As shown in Table 2, incretin drugs appear to dominate the current anti-obesity drug market and are the subject of numerous ongoing clinical trials. Novel anti-obesity therapeutics, including dual and especially triple incretin-based co-agonists, have for the first time allowed obese individuals to achieve very promising results in body weight loss, which tend to be comparable to those after bariatric surgery procedures, without exposure to this invasive treatment. Such a significant body weight reduction may slow down or even prevent the development of obesity-related comorbidities such as T2DM, and in the case of their coexistence it may contribute to the alleviation of the diseases, especially considering that a large number of the discussed drugs display independent glycemic control improvement. What seems optimistic is the fact that pharmacotherapy for obesity is currently a rapidly developing field of medicine. It is not always possible to clearly delineate the major mechanism of action of the specific compound, but based on current findings it seems that the most significant weight reduction, which is accompanied by an acceptable safety profile, is connected with a multifactorial approach. Incretin-based therapies seem to cover all major culprits in the development of obesity (brain, gut, and energy expenditure). This feature is abundantly seen in incretin mimetics. However, combined therapy with currently available drugs seems to be currently underexplored as a therapeutic option and, in our opinion, requires more research efforts. Novel potential treatment methods based on various pathomechanisms and different molecular points of application are being studied. Those compounds include impacts on hunger/satiety and the body’s energy expenditure. We are eagerly awaiting the results of those experiments due to the increasing burden of obesity and scarce therapeutic options.

## Figures and Tables

**Table 1:** Currently available weight-loss medications.

Drug	Approval Date	Mechanism of Action	Route of Administration	Dose	Contraindications	Common Side Effects
Orlistat [143]	FDA 1999 EMA 1998	Lipase inhibitor [143]	Oral	3 × 120 mg/day [143]	Chronic malabsorption syndrome, cholestasis, pregnancy [143]	Oily/fatty feces, bloating, fecal urgency [143]
Phentermine/topiramate [144]	FDA 2012	Serotonin-, norepinephrine-, and dopamine-releasing agent; GABA agonist [144]	Oral	From 3.75 mg/23 mg daily for 2 weeks (starting dose) to recommended 7.5 mg/46 mg, maximum dose: 15 mg/92 mg [144]	Glaucoma, pregnancy, hyperthyroidism, hypersensitivity to sympathomimetics, pregnancy [144]	Dizziness, insomnia, constipation [144]
Naltrexone/bupropion [60]	FDA 2014 EMA 2015	Opioid antagonist/ norepinephrine and dopamine reuptake inhibitor [60]	Oral	8 mg/90 mg daily (1 tablet), dose increased each week until maintenance dose: 2 tablets twice a day [60]	Uncontrolled hypertension, eating disorders (anorexia, bulimia), opioids use, seizure disorders, pregnancy [60]	Nausea, vomiting, constipation, diarrhea, dizziness [60]
Liraglutide [145] Semaglutide [146]	FDA 2014 EMA 2015 FDA 2021 EMA 2021	GLP-1 receptor agonists	Subcutaneous	Liraglutide: 0.6 mg daily (starting dose) increased weekly by 0.6 mg, target dose 3 mg [145] Semaglutide: from 0.25 mg once a week to full dose of 2.4 mg once a week [146]	Personal or family history of medullary thyroid carcinoma or in patients with multiple endocrine neoplasia syndrome type 2, acute pancreatitis, serious hypersensitivity, pregnancy [145,146]	Nausea, vomiting, constipation, diarrhea, abdominal pain [145,146]
Tirzepatide [140]	FDA 2023 EMA 2024	Dual GLP-1 and GIP receptor agonist	Subcutaneous	Initial dose: 2.5 mg once weekly, increasing in 2.5 mg increments after at least 4 weeks; recommended maintenance dose: 5/10/15 mg, max. 15 mg [140]	Personal or family history of medullary thyroid carcinoma or in patients with multiple endocrine neoplasia syndrome type 2, serious hypersensitivity [140]	Nausea, diarrhea, vomiting, constipation, abdominal pain, dyspepsia, injection site reactions, fatigue [140]
Setmelanotide [147]	FDA 2020 EMA 2021	MC4 receptor agonist	Subcutaneous	Starting dose: 2 mg/daily for 2 weeks, further depending on drug tolerance [147]	Prior serious hypersensitivity to setmelanotide [147]	Injection site reaction, skin hyperpigmentation, nausea, diarrhea, headache [147]
Metreleptin [148]	FDA 2014 EMA 2018	Leptin analogue	Subcutaneous	Females > 40 kg: initial 5 mg, max. 10 mg/daily, males > 40 kg: initial 2.5 mg, max. 10 mg/daily [148]	General obesity, severe hypersensitivity reactions [148]	Headache, hypoglycemia, abdominal pain [148]

**Table 2:** The currently pursued pharmacological interventions in obesity (according to https://clinicaltrials.gov/about-site/about-ctg web-base, accessed on 31 May 2024).

Mechanism of Action	Clinical Trial Phase	Compound Names	Number of Compounds According to Mechanism of Action (%)	Reference
Incretin-Based Therapies (41.57%)				
GLP-1 receptor agonists	I	ZT002	16 (17.98%)	NCT06371326
	I	CT-996		NCT05814107
	I	XW-004		NCT05184322
	II	Lotiglipron (PF-07081532)		NCT05579977
	II	Danuglipron (PF-06882961)		NCT04617275
	II	Noiiglutide (SHR2004)		NCT04799327
	II	GSBR-1290		NCT05762471
	II	HDM1002		NCT06500299
	II	HRS-7535		NCT06250946
	II	PB-119		NCT06350812
	II	RGT001-075		NCT06277934
	II	GZR-18		NCT06256562
	III	Ecnoglutide (XW-003)		NCT05813795
	III	Orforglipron (LY3502970)		NCT05869903
	III	HM11260C		NCT06174779
	III	TG103		NCT05997576
GLP-1 + GIP receptor agonists	II	Olatorepatide (HS-20094)	6 (6.74%)	NCT06118021
	II	RAY1225		NCT06254274
	II	HRS9531		NCT06054698
	II	VK2735		NCT06068946
	II	NN0519-0130		NCT06326060
	III	HRS9531		NCT06396429
GLP-1 + glucagon receptor agonists	II	Pemvidutid (ALT-801)	4 (4.49%)	NCT05295875
	II	PB-718		NCT06147544
	III	Mazdutide (IBI362; LY3305677)		NCT06164873
	III	Survodutide (BI 456906)		NCT06077864
GLP-1 receptor agonist + GIP receptor antagonist	II	Cafraglutide (AMG 133)	1 (1.12%)	NCT05669599
GLP-1 + GIP + glucagon receptor agonists	I	BI 3034701 (receptor specificity not yet disclosed)	2 (2.25%)	NCT06352437
	III	Retatrutide (LY3437943)		NCT06383390
GLP-1 + glucagon + FGF21 receptor agonist	I	DR10624	1 (1.12%)	NCT05378893
GLP-1 + GLP-2 receptor agonist	III	Dapiglutide	1 (1.12%)	NCT05788601
GLP-1 + amylin receptor agonists	II	Amycretin (NNC0487-0111)	2 (2.25%)	NCT06064006
	III	CagriSema (cagrilintide + semaglutide)		NCT06131437
Amylin analogues	I	Petrelintide (ZP8396)	4 (4.49%)	NCT05613387
	I	GUB014295		NCT06144684
	I	AZD6234		NCT06132841
	II	Eloralintide (LY3841136)		NCT06230523
Central Nervous System-Targeted Therapies (19.10%)				
Leptin receptor activators	I	ERX1000	2 (2.25%)	NCT04890873
	II	Mibavademab		NCT06373146
MC4R agonists	I	RM-718	2 (2.25%)	NCT06239116
	II	LB54640		NCT06046443
NPY receptor agonists	I	Nisotirostide (LY3457263)	3 (3.37%)	NCT05582096
	I	BI 1820237		NCT05751226
	II	NNC0165-1875		NCT04969939
Oxytocin analogues	II	Oxytocin (TNX-1900)	2 (2.25%)	NCT05664516
	III	Carbetocin		NCT06420297
GPR40 agonists	II	K-757	2 (2.25%)	NCT05900531
	II	K-833		NCT06019559
Ghrelin-O-acyltransferase inhibitor	I	BI 1356225	1 (1.12%)	NCT04065295
CB1R antagonist	I	GFB-024	1 (1.12%)	NCT04880291
CB1R agonist	II	Nabilone	1 (1.12%)	NCT04801641
CB1R inverse agonist	II	Monlunabant (INV-202)	1 (1.12%)	NCT05891834
Dopamine receptor agonist	II	NG101	1 (1.12%)	NCT06500429
Complex mechanism	III	Sibutramine/topiramate XR	1 (1.12%)	NCT05209984
Gastrointestinal Tract-Targeted Therapies (15.73%)				
Orally administered compounds reducing ingestion	II	GLY-200	2 (2.25%)	NCT06259981
	II	Acarbose + orlistat		NCT04521751
Microbiota-associated approach	I	XEN-101	6 (6.74%)	NCT06417697
	I	Probiotics/fecal microbiota transplantation		NCT05076656
	II	Probiotic blend		NCT05676229
	II	Fecal microbiota transplantation		NCT05253768
	II	Akkermansia Muciniphila WST01		NCT04797442
	II	Labisia pumila standardized extract (SKF7^®^)		NCT04557267
Bile acid-based approach	I	Ileocolonic-release conjugated bile acid	2 (2.25%)	NCT05314374
	I	Spermine–bile acid		NCT05925920
Glabridin analogue	II	Vutiglabridin (HSG4112)	1 (1.12%)	NCT05197556
Liver-targeted approach	I	TLC-6740	2 (2.25%)	NCT05822544
	I	ASC-41		NCT04686994
Dietary branched-chain amino acids (BCAAs)	I	Low-BCAAs diet	1 (1.12%)	NCT04424537
Drugs Potentially Modifying Energy Expenditure (15.73%)				
Renin–angiotensin system	I	Angiotensin-(1-7)	14 (15.73%)	NCT03777215
β3-Adrenergic receptor agonists	I	Mirabegron		NCT03049462
β2-Adrenergic receptor agonist	I	Salbutamol		NCT06319183
PEGylated arginine deiminase	II	ADI-PEG20		NCT05829239
Sodium–glucose transport protein-2 (SGLT-2) inhibitor	II	Empagliflozin		NCT05885074
Phosphodiesterase-5 (PDE-5) inhibitor	II	Tadalafil		NCT04684589
Guanylate cyclase stimulator	II	Vericiguat		NCT06320951
Unknown	III	Triticum aestivum		NCT06496100
Liver-enriched antimicrobial peptide	Not Applicable	LEAP-2 Protein		NCT05603598
Potent PPARγ binder nutraceutical	Not Applicable	Maslinic acid		NCT06484543
Form of vitamin B3, NAD+ precursor	Not Applicable	Nicotinamide riboside		NCT06044935
Nerve stimulation	Not Applicable	Olfactory stimulation with distinct odors		NCT05472168
Unknown	Not Applicable	Raw whole almonds		NCT06413069
GLP-1 receptor agonist	Not Applicable	Semaglutide		NCT06390501
Other Therapeutic Targets (7.87%)				
Antioxidant therapy	II	Methylglyoxal (MGO)-lowering cocktail	1 (1.12%)	NCT05083546
Monoacylglycerol acyltransferase-2 (MGAT-2) inhibitor	II	S-309309	1 (1.12%)	NCT05247970
Bitter taste (TAS2) receptor agonist (taste perception)	II	Eliapixant (ARD-101)	1 (1.12%)	NCT05121441
Interleukin-22 (IL-22)	II	CK-0045	1 (1.12%)	NCT05712876
Unknown	II	Sodium pentaborate pentahydrate	1 (1.12%)	NCT05741606
SGLT-2 inhibitors	III	Dapagliflozin	2 (2.25%)	NCT06000462
	IV	Henagliflozin		NCT06216340
Total number			89 (100%)	

## Data Availability

No new data were created or analyzed in this study. Data sharing is not applicable to this article.

## Abbreviations

α-MSH	α-Melanocyte-stimulating hormone
Acaca/ACC	Acetyl-CoA carboxylase
ACC1	Acetyl-CoA carboxylase 1
ACOX1	Acyl-CoA oxidase 1
ACS	Acyl-CoA synthetase
ADHD	Attention deficit hyperactivity disorder
AgRP	Agouti-related peptide
AICAR	5-Aminoimidazole-4-carboxamide ribonucleotide
AMPK	AMP-activated protein kinase
ATP	Adenosine triphosphate
BAIBA	β-Aminoisobutyric acid
BAs	Bile acids
BAT	Brown adipose tissue
BCAAs	Branched-chain amino acids
BD-AcAc2	R,S-1,3-Butanediol diacetoacetate
BDNF	Brain-derived neurotrophic factor
BED	Binge-eating disorder
BMI	Body mass index
BMPs	Bone morphogenetic proteins
C/EBPs	CCAAT/enhancer-binding proteins
CART	Cocaine- and amphetamine-regulated transcript
CB1R/CB2R	Cannabinoid-1 receptor/cannabinoid-2 receptor
CCK	Cholecystokinin
CD36	Cluster of differentiation 36
CDCA	Chenodeoxycholic acid
ChREBP	Carbohydrate-responsive element-binding protein
CLA	Conjugated linoleic acid
CNS	Central nervous system
COVID-19	Novel human coronavirus disease
Cox4i1	Cytochrome C oxidase subunit 4 isoform 1
CPT1	Carnitine palmitoyltransferase 1
CPT1b	Carnitine palmitoyltransferase 1b
CRH	Corticotropin-releasing hormone
DGAT1	Diacylglycerol O-acyltransferase 1
DHA	Docosahexaenoic acid
Dio2	Iodothyronine deiodinase 2
DNA	Deoxyribonucleic acid
EMA	European Medicines Agency
EPA	Eicosapentaenoic acid
F/B	Firmicutes/Bacteroidetes ratio
FABP3	Fatty acid-binding protein 3
FA	Fatty acid
FAS	Fatty acid synthase
FATP	Fatty acid transport protein
FDA	Food and Drug Administration
FFAR2	Free fatty acid receptor 2
FFAR3	Free fatty acid receptor 3
FGF21	Fibroblast growth factor 21
FIAF	Fasting-induced adipose factor
FMT	Fecal microbiota transplantation
FO	Fish oil
FXR	Farnesoid X receptor
GABA	Gamma-aminobutyric acid
GBA	Gut–brain axis
GHS-R1a	Growth hormone secretagogue receptor 1a
GI	Gastrointestinal
GIP	Glucose-dependent insulinotropic polypeptide
GLP-1	Glucagon-like peptide 1
GLP-1RA	GLP-1 receptor agonist
GLP-2	Glucagon-like peptide 2
GLUT4	Glucose transporter type 4
GPCRs	G-protein-coupled receptors
GPR40	G-protein-coupled receptor 40
HbA1c	Glycosylated hemoglobin
HCAR2	Hydrocarboxylic acid receptor 2
HDL	High-density lipoprotein
IKK2	Inhibitor of nuclear factor kappa-B kinase 2
IL-6	Interleukin-6
IL-22	Interleukin-22
JNK1 C	Jun N-terminal kinase 1
LCAD	Long-chain acyl-CoA dehydrogenase
LCFA-CoA	Long-chain fatty acyl-CoA
LEPR	Leptin receptor
LPL	Lipoprotein lipase
LPS	Lipopolysaccharide
Mapk14	Mitogen-activated protein kinase 14
MASH	Metabolic dysfunction-associated steatohepatitis
MASLD	Metabolic dysfunction-associated steatotic liver disease
MC3R	Melanocortin-3 receptor
MC4R	Melanocortin-4 receptor
MCAD	Medium-chain acyl-CoA dehydrogenase
MGAT-2	Monoacylglycerol acyltransferase-2
MGO	Methylglyoxal
NGPs	Next-generation probiotics
NPY	Neuropeptide Y
NPY5R	NPY receptor type 5
OXPHOS	Oxidative phosphorylation
PBEF	Pre-B-cell colony-enhancing factor
PDE-5	Phosphodiesterase-5
PGC-1α	Peroxisome proliferator-activated receptor gamma coactivator 1-alpha
POMC	Proopiomelanocortin
PP	Pancreatic polypeptide
PPARα/γ	Peroxisome proliferator-activated receptor alpha/gamma
PPARγ2	Peroxisome proliferator-activated receptor gamma 2
PRDM16	PR domain containing 16
PXR	Pregnane X receptor
PYY	Peptide YY
RBP4	Retinol-binding protein 4
RCT	Randomized controlled trial
RNA	Ribonucleic acid
SCD1	Stearoyl-CoA desaturase 1
SCFA	Short-chain fatty acid
SD	Standard deviation
SERCA	Sarco/endoplasmic reticulum Ca2+-ATPase
SF-1	Steroidogenic factor -1
SGLT-2	Sodium–glucose transport protein-2
SIRT-1	Sirtuin 1
SLC27A1	Solute carrier family 27 member 1 (another name for FATP)
SNAC	Salcaprozate sodium
SREBP-1c	Sterol-regulatory element-binding protein 1c
SST	Somatostatin
TAG	Triacylglycerol
T2DM	Type 2 diabetes mellitus
TLR4	Toll-like receptor 4
TNF-α	Tumor necrosis factor-α
TRH	Thyrotropin-releasing hormone
UCP1	Uncoupling protein 1
VDR	Vitamin D receptor
VIP	Vasoactive intestinal peptide
VLDL	Very-low-density lipoprotein
WAT	White adipose tissue
WHO	World Health Organization
